# P2X7 Receptor Antagonist Attenuates Retinal Inflammation and Neovascularization Induced by Oxidized Low-Density Lipoprotein

**DOI:** 10.1155/2021/5520644

**Published:** 2021-08-19

**Authors:** Mingzhu Yang, Ruiqi Qiu, Weiping Wang, Jingyang Liu, Xiuxiu Jin, Ya Li, Lei Li, Bo Lei

**Affiliations:** ^1^Henan Eye Institute, Henan Eye Hospital, People's Hospital of Zhengzhou University, Henan Provincial People's Hospital, Zhengzhou, Henan 450003, China; ^2^Xinxiang Medical University, Xinxiang, Henan 453003, China

## Abstract

Age-related macular degeneration (AMD) is a common and severe blinding disease among people worldwide. Retinal inflammation and neovascularization are two fundamental pathological processes in AMD. Recent studies showed that P2X7 receptor was closely involved in the inflammatory response. Here, we aim to investigate whether A740003, a P2X7 receptor antagonist, could prevent retinal inflammation and neovascularization induced by oxidized low-density lipoprotein (ox-LDL) and explore the underlying mechanisms. ARPE-19 cells and C57BL/6 mice were treated with ox-LDL and A740003 successively for i*n vitro* and *in vivo* studies. In this research, we found that A740003 suppressed reactive oxygen species (ROS) generation and inhibited the activation of Nod-like receptor pyrin-domain protein 3 (NLRP3) inflammasome and nuclear factor-*κ*B (NF-*κ*B) pathway. A740003 also inhibited the generation of angiogenic factors in ARPE-19 cells and angiogenesis in mice. The inflammatory cytokines and phosphorylation of inhibitor of nuclear factor-*κ*B alpha (IKB*α*) were repressed by A740003. Besides, ERG assessment showed that retinal functions were remarkably preserved in A740003-treated mice. In summary, our results revealed that the P2X7 receptor antagonist reduced retinal inflammation and neovascularization and protected retinal function. The protective effects were associated with regulation of NLRP3 inflammasome and the NF-*κ*B pathway, as well as inhibition of angiogenic factors.

## 1. Introduction

Age-related macular degeneration (AMD) is a blinding disease among people over the age of 50 worldwide. The prevalence of AMD is expected to increase dramatically as the global population ages [1]. AMD is closely related to chronic inflammation in retinal pigmented epithelial (RPE) cells, Bruch membrane, and choroid membrane [2]. There are two types of AMD according to the clinical characteristics, dry AMD and wet AMD. Dry AMD, also called nonexudative AMD, is characterized by retinal inflammation, which mainly happens in the early stage of AMD. Wet AMD, also called exudative AMD, is characterized by choroidal neovascularization and retinal angiogenesis, which usually happens in the late stage of AMD. Better understanding of the pathogenesis of AMD is crucial for seeking better and earlier treatment modalities.

The total cholesterol, triacylglycerol, and low-density lipoprotein (LDL) are higher in AMD patients compared to normal people [3]. Moreover, LDL is susceptible to oxidation, resulting in the formation of ox-LDL. ox-LDL can induce a large amount of ROS and oxidative stress injury. Chronic oxidative stress contributes to the pathogenesis of AMD. ox-LDL leads to lipid deposition, inflammation, and pyroptosis when it is accumulated in cells. It is reported that high level of ox-LDL may lead to oxidative stress and inflammation in glaucoma patients, which impedes wound healing after surgery [4]. The RPE becomes vulnerable to apoptosis through a number of mechanisms including the high oxidative stress environment. The interaction and uptake of ox-LDL are mediated by scavenger CD36 or AIM/CD5L in tissue macrophages [5, 6]. ox-LDL is also internalized by RPE and alters photoreceptor turnover and lysosomal function. The oxysterols in ox-LDL are cytotoxic to RPE cells [7]. It is found that ox-LDL induces a pathologic response in RPE, which suggests that ox-LDL is one trigger for initiating early events in the pathogenesis of AMD [8].

Retinal angiogenesis may also relate to the overproduction of ox-LDL and ROS generation. Recently, it is reported that ox-LDL is immunohistochemically detected in surgically excised choroidal neovascular membranes from eyes with AMD [9]. ox-LDL levels were higher in peripheral blood of AMD patients with choroidal neovascularization compared to normal individuals [10], which suggested a close relationship between ox-LDL and AMD. ox-LDL may also induce retinopathy through damaging the blood capillary [11]. These researches suggest that ox-LDL is highly related with retinal inflammation and neovascularization.

Pyroptosis related with NLRP3 inflammasome activation is recently identified to be a novel type of programmed cell death. The canonical inflammasome complexes are assembled around protein members of the nod-like receptor (NLRs) and composed of apoptosis-associated speck-like protein containing a caspase recruitment domain (ASC), converting the pro-Caspase-1 zymogen into a catalytically active enzyme [12]. NLRP3 inflammasome senses and responds to a diversity of pathogens or danger-associated molecular patterns. Overproduction of ROS activates NLRP3, recruiting ASC and regulating autoactivation of pro-Caspase-1. After activation of pro-Caspase-1, pro-IL-1*β* and pro-IL-18 are cleaved into mature peptides and secreted outside to mediate the following inflammation. Activation of the NF-*κ*B pathway upregulates the transcription of NLRP3 and pro-IL-1*β*. ox-LDL induced a large amount of ROS in macrophage cells and activated NLRP3 inflammasome and then promoted the secretion of inflammatory cytokines such as IL-1*β* and IL-18. Besides, the ROS mediated by ox-LDL also leads the activation of the NF-*κ*B signaling pathway, which may strengthen the inflammation response [12].

The P2X7 receptor (P2X7R) is an ATP-gated ion channel which is a key player in oxidative stress under pathological conditions [13]. P2X7R is expressed in RPE and neural retina [14]. Overactivation of P2X7R plays a pathogenic role in age-related photoreceptor cell death and degeneration of the retinal pigment epithelium [15]. Activation of P2X7R induces calcium ion influx and drives NLRP3 inflammasome activation and IL-1*β* processing and release [16]. P2X7R agonist treatment led to increased expression of proinflammatory mediators (IL-1*β*, etc.), P2X7R, and VEGF-A, along with enhanced production of ROS [17]. Carbon monoxide (CO) is an endogenous gasotransmitter that limits inflammation and prevents apoptosis in several areas including the eye. Incidentally, CO is implicated in optic neuropathy. Low concentrations of CO are cytoprotective in neurons and microglia, and P2X7R could modulate CO production [18]. P2X7 receptor-mediated NLRP3 inflammasome activation always happened in many pathophysiologic processes such as diabetic retinopathy [19]. In addition, autocrine purinergic signaling mediated by a release of ATP, activation of P2X7 receptor and NF-*κ*B, is required for the full expression of NFAT5 gene under diabetic retinopathy [20, 21]. These researches suggest that there is a huge correlation between P2X7 receptor and age-related retinal diseases. A740003, an effective P2X7R antagonist, is commonly used in previous studies. Recently, it is reported that P2X7R antagonist protects retinal ganglion cells by inhibiting microglial activation [22, 23]. P2X7R blockade fully reversed retinal vascular permeability increase, VEGF accumulation, and IL-6 expression [24]. Therefore, we speculated that P2X7R inhibitor attenuated retinal inflammation induced by ox-LDL through regulating the activation of the NF-*κ*B pathway and NLRP3 inflammasome and reduced retinal angiogenesis by downregulating the expression of VEGF and HIF-1*α*.

## 2. Materials and Methods

### 2.1. Preparation of ox-LDL and A740003

Human ox-LDL was purchased from Solarbio Science & Technology Co., Ltd (Beijing, China). ARPE-19 cells were exposed to 100 *μ*g/ml ox-LDL diluted with DMEM/F12 medium. A concentration of 3.0 mg/ml ox-LDL was used for subretinal injection of C57BL/6 mice. A740003 (ApexBio, Huston, TX, USA) was dissolved in 100% dimethyl sulfoxide (DMSO) and diluted with DMEM/F12 medium or PBS to a final DMSO concentration no more than 1%. DMEM/F12 dilution was used for ARPE-19 treatment, and PBS dilution was used for intraperitoneal injection of C57BL/6 mice.

### 2.2. Cell Culture and Treatment

ARPE-19 cells were purchased from the American Type Culture Collection (ATCC, Manassas, VA, USA). ARPE-19 cells (1.5 × 10^5^ cells/ml) were grown in DMEM/F12 medium (Gibco, New York, NY, USA) supplemented with 10% FBS (TBD science, Tianjin, China), 1% penicillin, and streptomycin (Solarbio) in 37°C with 5% CO_2_ incubator. The optimal ox-LDL concentration for the following studies was 100 *μ*g/ml, which was chosen based on the mRNA expression levels of NLRP3 and VEGF. The optimal concentration is corresponding to the previous report [25]. ARPE-19 cells were exposed to 100 *μ*g/ml of ox-LDL for 24 hours with 2-hour pretreatment of 1 *μ*M A740003. Besides, ARPE-19 cells were pretreated with 200 *μ*M of A740003 for 2 hours, followed by stimulation of 100 *μ*g/ml ox-LDL for 48 hours. The optimal concentrations of A740003 for 24/48 hours exposure to ox-LDL were screened by quantitative real-time PCR (Figure S1).

### 2.3. Animal Care and Use

Male C57BL/6 mice were purchased from Jackson Laboratories (Bar Harbor, ME, USA). The animals were housed under 12 h light-dark cycle and given a standard chow diet. Animal care followed the guidelines formulated by the Association for Research in Vision and Ophthalmology (ARVO). Experiments and procedures involving animals were permitted by the Ethics Committee of Henan Eye Institute. Every effort was made to minimize animal discomfort and stress.

Mice of 6~8-week-old were randomly divided into four groups: (1) control group: treated with subretinal injection of 1 *μ*l PBS (Solarbio); (2) ox-LDL group: treated with subretinal injection of 1 *μ*l ox-LDL (3.0 mg/ml) (Sigma-Aldrich, St. Louis., MO, USA); (3) ox-LDL+vehicle group: treated with subretinal injection of 1 *μ*l ox-LDL (3.0 mg/ml). 1% DMSO in PBS served as vehicle and was intraperitoneally injected daily from day 3 before to day 14 after ox-LDL subretinal injection; and (4) ox-LDL+A740003 group: treated with subretinal injection of 1 *μ*l ox-LDL (3.0 mg/ml). A740003 (30 mg/kg body weight/day) was intraperitoneally injected daily from day 3 before to day 14 after ox-LDL injection. Two weeks after subretinal injection of ox-LDL, mice were sacrificed for the following experiments.

### 2.4. Subretinal Injection of ox-LDL

One *μ*l ox-LDL (3.0 mg/ml) was injected subretinally into the right eyes of the mice, and the lateral eyes were not injected. Injection was performed according to a protocol described previously [26]. Briefly, mice were anesthetized with intraperitoneal injection of a mixture of ketamine (75 mg/kg; Fujian Gutian Pharmaceutical Co., Ltd., Ningde, China) and xylazine (13.6 mg/kg; VEDCO, Inc., St. Joseph, MO, USA) diluted in saline. Pupils were dilated with tropicamide phenylephrine eye drops (Santen Pharmaceutical Co., Ltd, Osaka, Japan) 10 minutes prior to injection. An aperture was made through the sclera, below the ora serrata with a 30-gauge needle. Then, a blunt 32-gauge Hamilton syringe was inserted through the aperture, avoiding damage of the lens and penetrating the neuroretina. One *μ*l ox-LDL was injected into the subretinal space under the dissecting microscope. Successful delivery of ox-LDL was confirmed by viewing subretinal blebs demarcating the retinal detachment in the injected retinal area. Such detachments usually resolved within 1 to 2 days. All animals received antibiotic eye drops on the cornea and were observed daily after operation. Only animals with minimal surgical complications and initial retinal blebs occupying more than 60% of the retina were retained for further study.

### 2.5. Real-Time Quantitative PCR Analysis

ARPE-19 cells in 12-well plates were collected by digestion of 0.25% trypsin and centrifuging. Eyeballs of mice were enucleated at 2 weeks after ox-LDL subretinal injection. Retinas were dissected and homogenized for total RNA extraction. The mRNA levels of NLRP3, Caspase-1, P2X7R, VEGF, and HIF-1*α* in ARPE-19 cells and retinas were detected by qPCR assay. Total RNA was extracted with Trizol reagent (ThermoFisher Scientific, Waltham, MA, USA) from ARPE-19 cells and retinas according to the manufacturer's instructions. Complementary DNA (cDNA) was generated by using the PrimeScript® RT reagent kit (Takara Biotechnology, Dalian, China). qPCR was performed according to the manufacturer's instructions with the ABI Prism 7500 system (Applied Biosystems, Foster City, CA, USA). The amplification system used for qPCR was a volume of 20 *μ*l PowerUp™ SYBR® Green Master Mix (ThermoFisher Scientific). The cycling protocol comprised of 50°C for 2 minutes and then 95°C for 2 minutes, followed by 40 cycles at 95°C for 15 seconds and 60°C for 1 minute. To determine the mRNA expression, which was normalized to the endogenous reference gene *β*-actin, all samples were detected in triple. Relative quantification was achieved by the comparative 2^-*ΔΔ*Ct^ method. The sequences of primers used for qPCR assay are shown in Table 1 and Table 2.

### 2.6. Enzyme-Linked Immunosorbent Assay

After pretreatment with 1 *μ*M A740003 for 2 hours, ARPE-19 cells were induced with ox-LDL for 24 hours. Then, supernatants of all groups were collected and centrifuged to detect the concentrations of IL-1*β* (RayBiotech, Norcross, GA, USA) and IL-18 (R&D Systems, Minneapolis, CA, USA) by using human ELISA kits according to the manufacturers' protocols. The concentrations of IL-1*β* and IL-18 were calculated according to optical density measured at 450 nm by subtracting the optical density measured at 540 or 570 nm using a multifunction microplate reader (PerkinElmer, Waltham, MA, USA).

### 2.7. Reactive Oxygen Species (ROS) Assay and ATP Assay

ARPE-19 cells were divided into four groups: (1) control group: incubated with DMEM/F12 basal medium; (2) ox-LDL group: exposed to 100 *μ*g/ml ox-LDL; (3) ox-LDL+vehicle group: pretreated with DMSO for 2 hours and then exposed to 100 *μ*g/ml ox-LDL; and (4) ox-LDL+A740003 group: pretreated with 1 *μ*M A740003 for 2 hours and then exposed to 100 *μ*g/ml ox-LDL. 24 hours later after exposure to ox-LDL, ARPE-19 cells were subjected to ROS assay according to the manufacturer's instruction (Beyotime, Shanghai, China). Briefly, ARPE-19 cells were exposed to 10 *μ*M DCFH-DA probe for 30 minutes at 37°C and then washed with DMEM/F12 basal medium for 3 times. Fluorescence of DCF was detected by a fluorescent microscope at 488 nm excitation wavelength and 525 nm emission wavelength. The fluorescent intensity was indicated by the average optical density. The average optical density of each group was measured using Image-Pro Plus 6.0 software (Media Cybernetics, Inc., Rockville, MD, USA). ATP content in ARPE-19 cells was determined by the Enhanced ATP Assay Kit (Beyotime, Shanghai, China) according to the manufacturer's instructions, and the results are shown in Figure S2.

### 2.8. Western Blot Analysis

ARPE-19 cells in 6-well plates were washed with PBS (Solarbio) for three times and lysed with RIPA lysis buffer for WB/IP assays (Yesen, Shanghai, China) containing 1% protease inhibitor cocktail (ApexBio, Houston, TX, USA) on the ice for 30 minutes. Similarly, retinas were dissected from eyeballs of mice and homogenized with lysis buffer containing 1% protease inhibitor cocktail on the ice for 30 minutes. The supernatants were collected after centrifuging the cell lysate at 12,000 rpm for 15 minutes. The protein concentration was detected by using bicinchoninic acid (BCA) protein kit (Beyotime). All samples were diluted with 5× SDS loading buffer (EpiZyme, Shanghai, China) and boiled at 100°C for 5 minutes. Equal amounts of total protein were separated on a 10% SDS-polyacrylamide gel and transferred to polyvinylidenedifluoride (PVDF) membranes (Millipore Corporation, Burlington, MA, USA). After blocking with 5% nonfat milk for 1.5 hours, the membranes were incubated with specific primary antibodies against NLRP3 (1 : 100, Cell Signaling Technology, Danvers, MA, USA), pro-Caspase-1 and Caspase-1 (1 : 200, Abcam, Cambridge, MA, USA), P2X7R (1 : 1000, Novus Biologicals, Littleton, CO, USA), VEGFA (1 : 450, Abcam), HIF-1*α* (1 : 1000, Novus), p-I*κ*B-*α* (1 : 500, Abcam), I*κ*B-*α* (1 : 1000, Abcam), IL-1*β* (1 : 500, Abcam), and *β*-actin (1 : 1000, Abcam) overnight at 4°C. After washing, the membranes were incubated with secondary antibody (1 : 10000, Millipore) at room temperature for 2 hours under 50 rpm gently shaking. Signals were developed with ECL kit (Millipore), and band densitometry was performed using the AlphaView SA Software (ProteinSimple, San Jose, CA, USA). *β*-Actin was used as loading control. Measurements were repeated three times for each experiment.

### 2.9. ERG Assessment

Retinal function was assessed with ERG following a previously described procedure [27]. After overnight dark adaptation, mice were anesthetized as previously described. The pupils were dilated with tropicamide eye drops 30 minutes prior to recording. Needle electrodes were subcutaneously inserted into the back and the tail as reference and ground leads, respectively. Active electrodes were gently positioned on the center of the cornea. All procedures were performed under dim red light. Full-field ERGs were recorded with RetiMINER-C, a visual electrophysiology system (AiErXi Medical Equipment Co., Ltd., Chongqing, China). A series of stimulus intensities ranging from -3 to 1 log cd-s/m^2^ was applied for dark-adapted ERGs. After light adaptation of 5 minutes, light-adapted ERGs were recorded to strobe-flash stimuli (0 and 1 log cd-s/m^2^) superimposed on the background light. Responses to brief flashes were analyzed by measuring the amplitudes of the a- and b-waves.

### 2.10. Whole Flat Mount of Mouse Retinas

Retinal whole flat mounts were prepared as previously described [28]. Briefly, the eyeballs from C57BL/6 mice were removed and fixed in 4% paraformaldehyde at room temperature for 1 hour. An incision to the cornea under a dissecting microscope was made. At incision, the sclera was peeled towards the optic nerve and then the lens and iris were removed. The retinas were extracted and permeabilized in 0.5%Triton X-100 for 2 hours. The retinas were stained by isolectin-B4 for 30 minutes by gently shaking. After staining, the retinas were cut at 3, 6, 9, and 12 o'clock for 4 incisions. Antifluorescence mounting media were used for resistance to fluorescence quenching before covering the retinas by a coverslip. Flat mounts were examined by fluorescence microscopy (Olympus, Tokyo, Japan). Isolectin-B4 was used for staining retinal vessels. The new growth tips indicating angiogenesis were counted in the same area (54000 *μ*m^2^) of fluorescent images.

### 2.11. Immunofluorescent Staining

Immunohistochemistry was performed using the methodology as previously described [27]. Eyes were enucleated at 2 weeks after subretinal injection of ox-LDL and fixed with 4% paraformaldehyde overnight. After dehydration, the eyes were embedded in melting paraffin. Serial 4 *μ*m paraffin sections were cut through the cornea-optic nerve axis. Tissue sections were subsequently treated for antigen retrieval and blocked with 5% BSA for 30 minutes. Then, sections were incubated with anti-VEGF antibody (1 : 200; Servicebio, Wuhan, China) overnight at 4°C. After washing, the slides were incubated with anti-rabbit secondary antibody for 50 minutes and DAPI for 10 minutes in the dark. Antifluorescence mounting media were used for resistance to fluorescence quenching. Paraffin sections were examined under a fluorescence microscope. The fluorescent intensity indicated by average optical density of each section was measured using Image-Pro Plus 6.0 software.

### 2.12. Statistical Analysis

Results from experiment were expressed as mean ± standard error of the mean (SEM). Statistical analysis was analyzed by the GraphPad Prism 7 software (GraphPad Software, San Diego, CA, USA). Experimental data were analyzed by one-way ANOVA or two-way ANOVA followed by Bonferroni correction. *p* value less than 0.05 was considered as statistically significant.

## 3. Results

### 3.1. A740003 Inhibited the Activation of NLRP3 Inflammasome and Phosphorylation of IKB*α* and Decreased the Expression of P2X7R in ARPE-19 Cells

**Figure 1:**
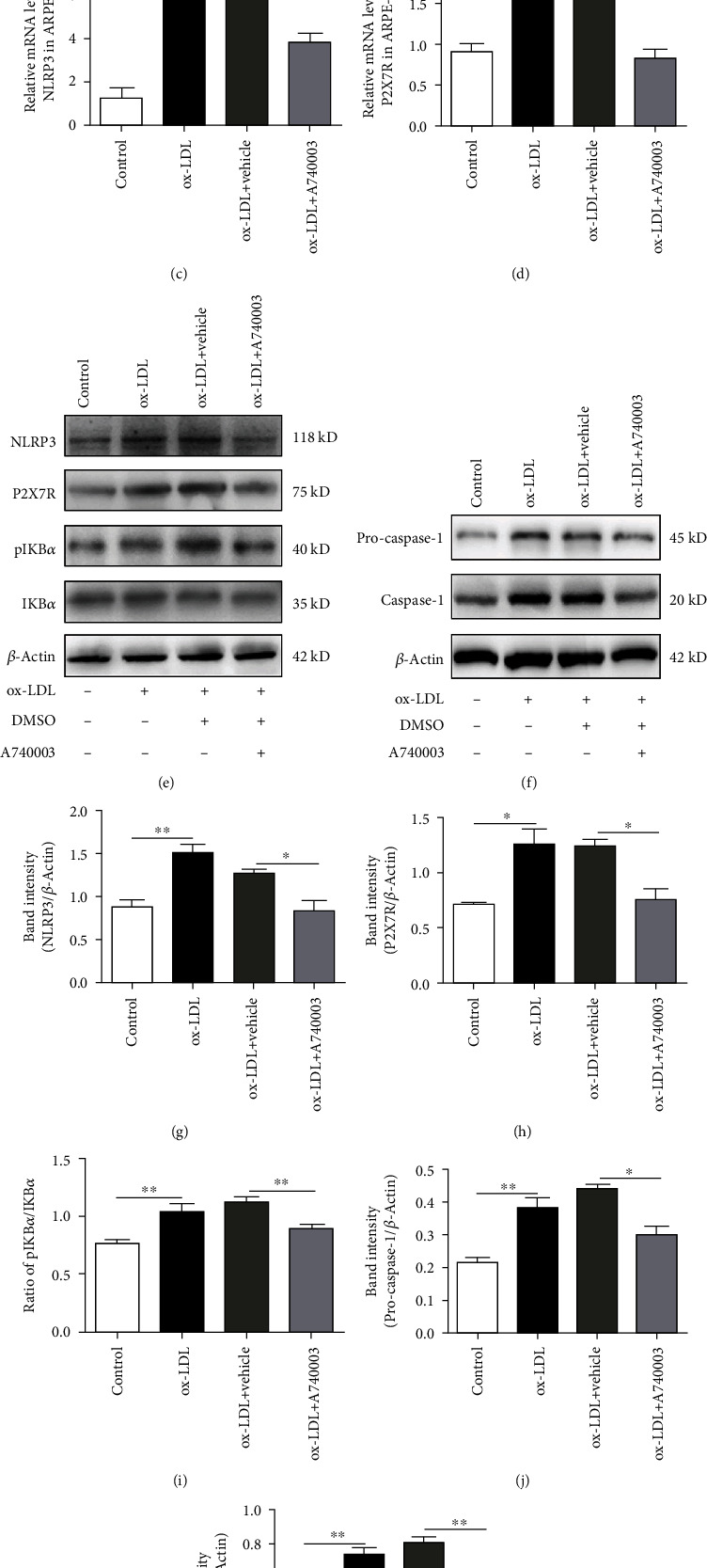
A740003 inhibited the activation of NLRP3 inflammasome and phosphorylation of IKB*α* and decreased the expression of P2X7R in ARPE-19 cells exposed to ox-LDL for 24 hours. ELISA results showed that A740003 pretreatment decreased the secretion of inflammatory cytokines including IL-1*β* (to 2.831 ± 0.1162) (a) and IL-18 (to 1.585 ± 0.2329) (b) significantly. Besides, compared to the vehicle-treated group, A740003 pretreatment significantly inhibited NLRP3 (56.73% ± 0.3908) (c) and P2X7R (51.30% ± 0.1196) (d) at mRNA levels in ARPE-19 cells. It indicated that A740003 inhibited the overexpression of P2X7R and NLRP3 which was induced by ox-LDL. Western blot showed that A740003 downregulated the protein levels of NLRP3 (64.92% ± 0.1264) (e, g), pro-Caspase-1 (68.26% ± 0.02456) (f, j), Caspase-1 (65.97% ± 0.01661) (f, k), and P2X7R (60.55% ± 0.09754) (e, h) and the phosphorylation of IKB*α* (79.34% ± 0.01995) (e, i) significantly compared to the vehicle-treated group. Symbols: +: with; −: without. The results were mean ± SEM. Significance of difference (^∗^*p* < 0.05, ^∗∗^*p* < 0.01, and ^∗∗∗^*p* < 0.001) was determined by using one-way ANOVA with Bonferroni correction.

ARPE-19 cells were cultured with 100 *μ*g/ml of ox-LDL for 24 hours. Secretion of inflammatory cytokines was determined by ELISA. ELISA results showed that ox-LDL promoted the secretion of IL-1*β* (Figure 1(a)) and IL-18 (Figure 1(b)) significantly compared to the control group. The upregulated secretion of IL-1*β* and IL-18 indicated that more pro-IL-1*β* and pro-IL-18 were cleaved to mature inflammatory cytokines under the effects of mature Caspase-1. Pro-Caspase-1 turned into mature Caspase-1 when NLRP3 inflammasome was activated. Therefore, the ELISA results confirmed the activation of NLRP3 inflammasome in ARPE-19 cells exposed to ox-LDL. It indicated that ox-LDL induced the activation of NLRP3 inflammasome. Moreover, the qPCR assay was performed to determine the mRNA levels of NLRP3 and P2X7R. ox-LDL upregulated the mRNA levels of NLRP3 and P2X7R significantly (Figures 1(c) and 1(d)). Western blot was performed to evaluate the levels of proteins related with NLRP3 inflammasome. The results showed that the protein levels of NLRP3 (Figures 1(e) and 1(g)), pro-Caspase-1 (Figures 1(f) and 1(j)) Caspase-1 (Figures 1(f) and 1(k)), and P2X7R (Figures 1(e) and 1(h)) and the phosphorylation of IKB*α* (Figures 1(e) and 1(i)) in ARPE-19 cells increased obviously after exposure to ox-LDL.

Furthermore, ARPE-19 cells were pretreated with 1 *μ*M A740003 for 2 hours before 24-hour ox-LDL incubation. ELISA results showed that A740003 pretreatment reversed the effects of ox-LDL incubation. It decreased the secretion of inflammatory cytokines including IL-1*β* (Figure 1(a)) and IL-18 (Figure 1(b)) significantly compared to the vehicle-treated group. In addition, compared to the vehicle-treated group, A740003 pretreatment decreased the mRNA levels of NLRP3 (Figure 1(c)) and P2X7R (Figure 1(d)) distinctly in ARPE-19 cells. A740003 inhibited the overexpression of P2X7R and NLRP3 which was induced by ox-LDL. The Western blot results showed that protein levels of NLRP3, Caspase-1, and P2X7R increased robustly in ARPE-19 cells exposed to ox-LDL. However, pretreatment with A740003 could remarkably downregulate the protein expression of NLRP3, P2X7R, pro-Caspase-1, and Caspase-1 in ARPE-19 cells (Figures 1(e)–1(h), 1(j), and 1(k)). The phosphorylation of IKB*α* was closely related with the activation of the NF-*κ*B signaling pathway. Western blot results also showed that ox-LDL enhanced the ratio of p-IKB*α* to IKB*α* protein significantly. Moreover, the phosphorylation of IKB*α* was inhibited significantly in ARPE-19 cells pretreated with A740003 (Figures 1(e) and 1(i)). In conclusion, the results suggested that the ox-LDL stimulation could upregulate the secretion of inflammatory cytokines, which indicated the activation of NLRP3 inflammasome and pro-Caspase-1. However, P2X7R antagonist could inhibit the expression and secretion of inflammatory cytokines induced by ox-LDL. Moreover, the activation of NLRP3 inflammasome and the NF-*κ*B signaling pathway induced by ox-LDL could be suppressed by A740003 treatment.

### 3.2. A740003 Suppressed the Upregulation of Angiogenic Growth Factors in ARPE-19 Cells Exposed to ox-LDL

**Figure 2:**
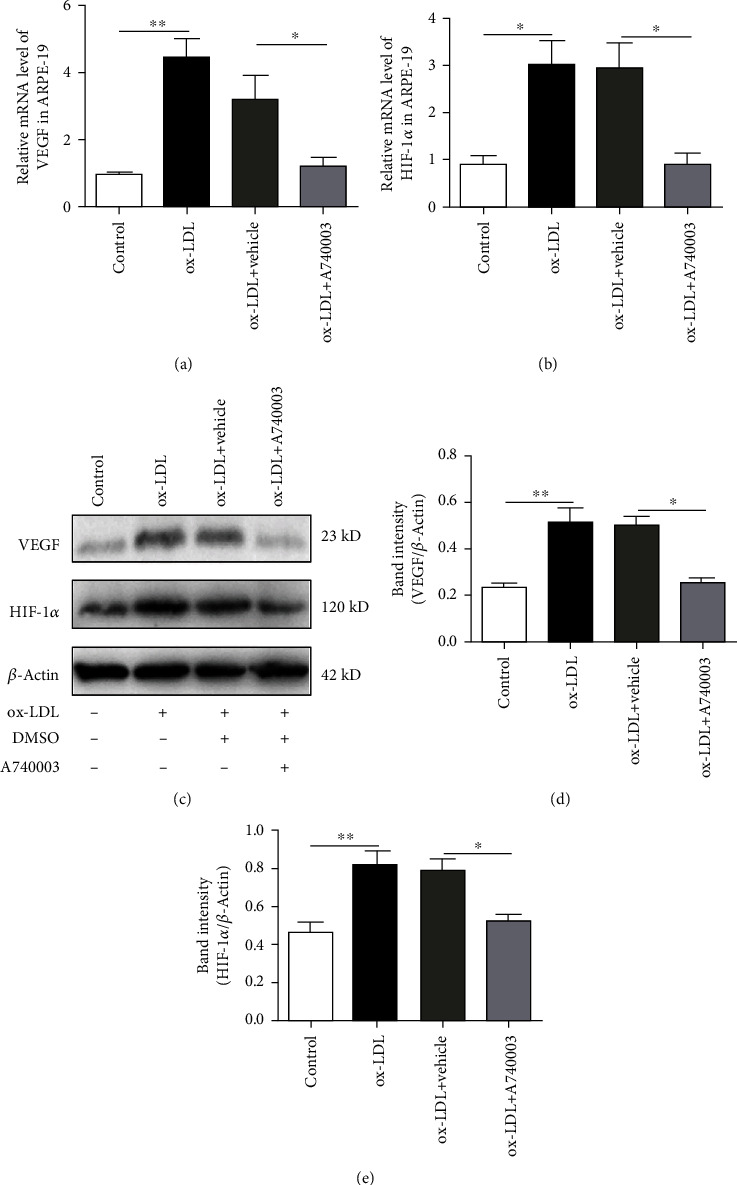
A740003 suppressed the upregulation of angiogenic growth factors in ARPE-19 cells induced by ox-LDL incubation for 48 hours. The mRNA levels of VEGF and HIF-1*α* were detected by qPCR. Compared to the vehicle-treated group, A740003 pretreatment significantly suppressed the mRNAs of VEGF (38.12% ± 0.2505) (a) and HIF-1*α* (30.28% ± 0.2455) (b) in ARPE-19 cells. Besides, the protein levels of VEGF and HIF-1*α* were detected by Western blot. A740003 pretreatment also decreased the overexpression of VEGF (51.03% ± 0.01724) (c, d) and HIF-1*α* (66.43% ± 0.03408) (c, e) compared to vehicle-treated cells. Symbols: +: with; −: without. The results were mean ± SEM. Significance of difference (^∗^*p* < 0.05, ^∗∗^*p* < 0.01, and ^∗∗∗^*p* < 0.001) was determined by using one-way ANOVA with Bonferroni correction.

ARPE-19 cells were treated with 100 *μ*g/ml of ox-LDL for 48 hours. The qPCR assay was performed to determine the mRNA levels of VEGF and HIF-1*α*. The results are shown in Figure 2; mRNA levels of VEGF and HIF-1*α* increased significantly in ARPE-19 cells exposed to ox-LDL (Figures 2(a) and 2(b)). VEGF and HIF-1*α* are primary angiogenic proteins contributing to neovascularization [29]. Thus, the protein levels of VEGF and HIF-1*α* are vital for evaluating the possibility of retinal neovascularization. Western blot was performed to detect the levels of proteins related with angiogenesis. The results demonstrated that the protein levels of VEGF and HIF-1*α* upregulated obviously in ARPE-19 cells incubated with ox-LDL (Figures 2(c)–2(e)).

In addition, another group of ARPE-19 cells was pretreated with 200 *μ*M A740003 for 2 hours before ox-LDL incubation. The qPCR and Western blot assay were performed to determine the mRNA and protein levels of HIF-1*α* and VEGF in ARPE-19 cells pretreated with A740003. The results manifested that mRNA levels of VEGF and HIF-1*α* decreased evidently in ARPE-19 cells pretreated with A740003 (Figures 2(a) and 2(b)). Furthermore, the protein levels of VEGF and HIF-1*α* were suppressed effectively by A740003 pretreatment in ARPE-19 cells (Figures 2(c)–2(e)). The results indicated that ox-LDL increased the mRNA and protein levels of angiogenic growth factors including VEGF and HIF-1*α* in ARPE-19 cells, whereas A740003 pretreatment could inhibit the HIF1*α*/VEGF axis effectively in ARPE-19 cells exposed to ox-LDL. A740003 might be effective to suppress the retinal neovascularization through inhibiting the HIF1*α*/VEGF axis.

### 3.3. A740003 Suppressed the ROS Generation Induced by ox-LDL in ARPE-19 Cells

**Figure 3:**
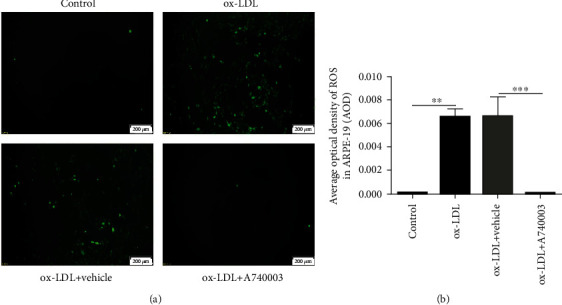
A740003 inhibited the ROS production in ARPE-19 cells induced by ox-LDL. The fluorescence of DCFH-DA increased robustly in ARPE-19 cells incubated with ox-LDL (a). The average optical density of ROS in the ox-LDL group was obviously higher than that in the control group (b). However, A740003 pretreatment could decrease the fluorescence of DCFH-DA in ARPE-19 cells induced by ox-LDL (a, b). The overproduction of ROS induced by ox-LDL was suppressed by A740003. The results were mean ± SEM. Significance of difference (^∗^*p* < 0.05, ^∗∗^*p* < 0.01, and ^∗∗∗^*p* < 0.001) was determined by using one-way ANOVA with Bonferroni correction.

ROS generation in ARPE-19 cells was detected by a DCFH-DA fluorescent probe and microscope after 24-hour exposure to ox-LDL. The results showed that DCFH-DA fluorescent intensity was significantly increased after ox-LDL incubation compared to the control group (Figure 3(a)). Image-Pro Plus 6.0 software was used to calculate the average optical density (AOD) of each group. The results showed that the average optical density in the ox-LDL incubated group was obviously higher than that in the control group (Figure 3(b)). It indicated that ROS generation was successfully induced by ox-LDL incubation in ARPE-19 cells. Moreover, compared to the vehicle-treated group, DCFH-DA fluorescent density in the A740003-treated group was significantly decreased, suggesting that ROS production was effectively suppressed by A740003 pretreatment (Figures 3(a) and 3(b)).

### 3.4. A740003 Inhibited the Activation of NLRP3 Inflammasome and Phosphorylation of IKB*α* and Decreased the Overexpression of P2X7R In Vivo

**Figure 4:**
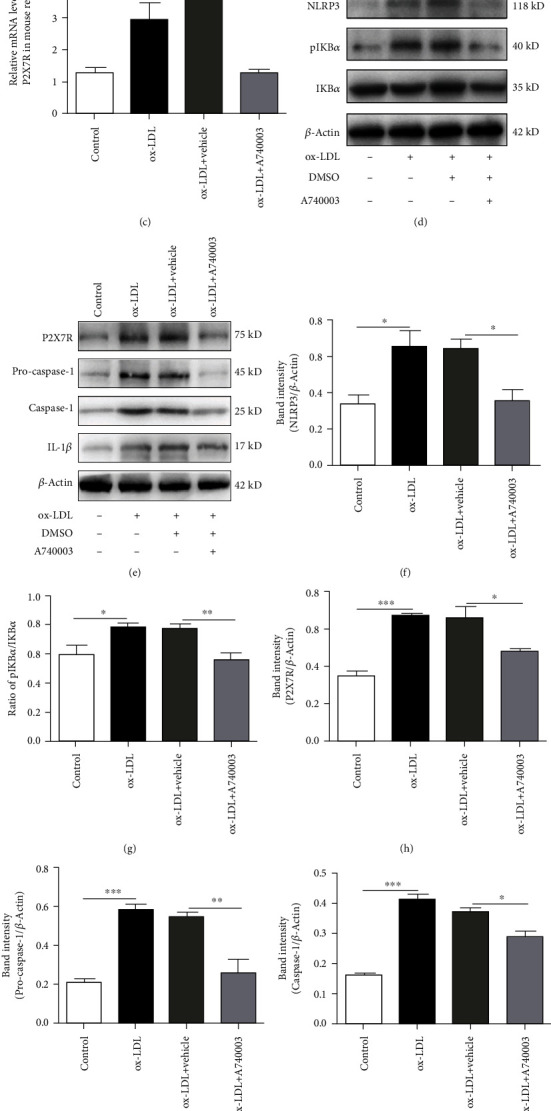
A740003 inhibited the activation of NLRP3 inflammasome and phosphorylation of IKB*α* and decreased the overexpression of P2X7R in C57BL/6 mouse retinas injected with ox-LDL. The qPCR showed that compared to the vehicle-treated group, intraperitoneal injection of A740003 reduced the mRNA expression of NLRP3 (29.03% ± 0.05369) (a), Caspase-1 (39.79% ± 0.3646) (b), and P2X7R (34.73% ± 0.09999) (c) in C57BL/6 mouse retina obviously. Moreover, Western blot showed that protein levels of NLRP3 (55.03% ± 0.06134) (d, f), pro-Caspase-1 (47.16% ± 0.06827) (e, i), Caspase-1 (78.19% ± 0.0134) (e, j), IL-1*β* (57.09% ± 0.03169) (e, k), and P2X7R (73.04% ± 0.006164) (e, h) in C57BL/6 mouse retina were inhibited by A740003 significantly. A740003 treatment also decreased the phosphorylation of IKB*α* (72.25% ± 0.04076) (d, g) in C57BL/6 mouse retina. Symbols: +: with; −: without. The results were mean ± SEM. Significance of difference (^∗^*p* < 0.05, ^∗∗^*p* < 0.01, and ^∗∗∗^*p* < 0.001) was determined by using one-way ANOVA with Bonferroni correction (*n* = 4-6).

C57BL/6 mice were subretinally injected with 1 *μ*l ox-LDL. A740003 (30 mg/kg body weight/day) was intraperitoneally injected daily from 3 days before ox-LDL injection. Two weeks later, qPCR and Western blot assay were performed to determine the mRNA and protein levels of NLRP3, P2X7R, and Caspase-1. The protein level of IL-1*β* and phosphorylation of IKB*α* were also assessed by Western blot. As shown in Figure 4, mRNA levels of NLRP3, Caspase-1, and P2X7R were upregulated significantly in mice injected with ox-LDL, whereas treatment with A740003 daily could inhibit the increase of NLRP3, Caspase-1, and P2X7R at mRNA level effectively compared to the vehicle-treated group (Figures 4(a)–4(c)). Furthermore, Western blot results demonstrated that protein levels of NLRP3, pro-Caspase-1, Caspase-1, IL-1*β*, and P2X7R increased remarkably in mouse retinas injected with ox-LDL (Figures 4(d)–4(f) and 4(h)–4(k)). It indicated that ox-LDL injection activated NLRP3 inflammasome. However, compared to the vehicle-treated group, the protein levels of NLRP3, pro-Caspase-1, Caspase-1, IL-1*β*, and P2X7R in mouse retinas of the A740003-treated group decreased significantly (Figures 4(d)–4(f) and 4(h)–4(k) ). Subretinal injection of ox-LDL could increase the phosphorylation of IKB*α* and thus induce the activation of the NF-*κ*B signaling pathway (Figures 4(d) an(d) 4(g)). The Western blot results also showed that the ratio of p-IKB*α* to IKB*α* in mouse retinas of the A740003-treated group significantly decreased compared to the vehicle-treated group (Figures 4(d) and 4(g)). The results showed that ox-LDL could activate the NLRP3 inflammasome and NF-*κ*B signaling pathway significantly. It indicated that A740003 treatment decreased the expression of P2X7R and suppressed the activation of the NLRP3 inflammasome and NF-*κ*B signaling pathway *in vivo*.

### 3.5. A740003 Suppressed the Expression of HIF-1*α* and VEGF in Mice Subretinally Injected with ox-LDL

**Figure 5:**
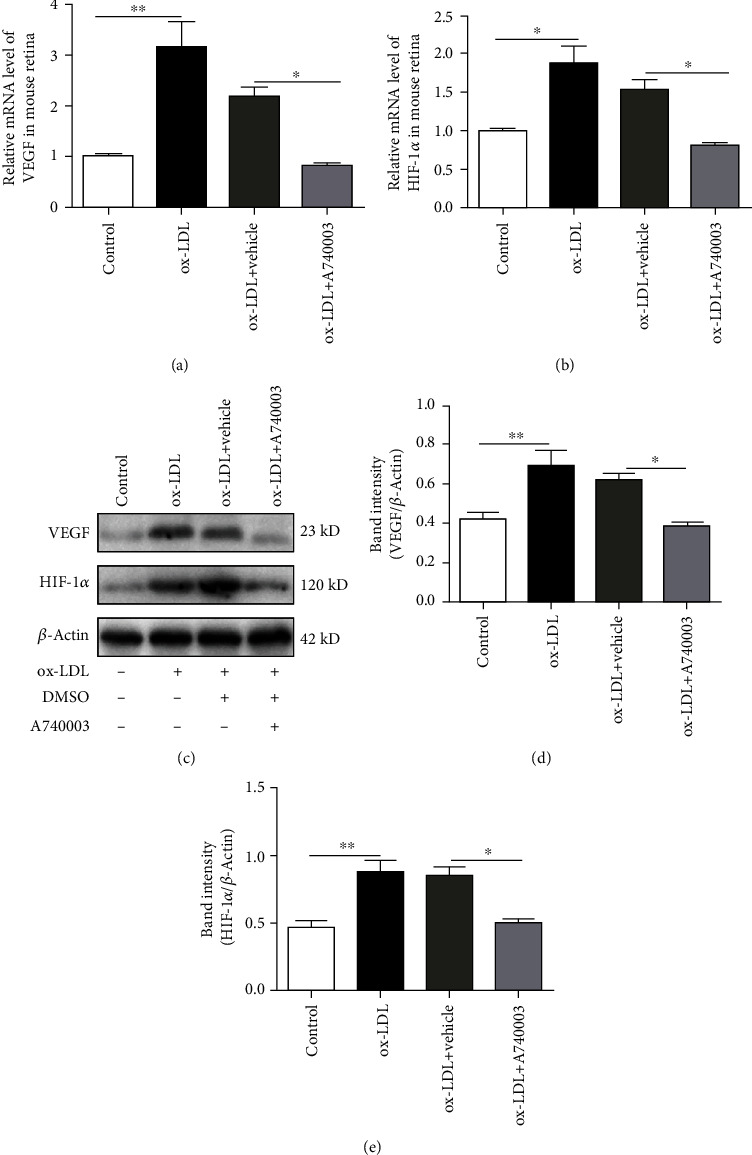
A740003 inhibited the upregulation of VEGF and HIF-1*α* in C57BL/6 mouse retinas injected with ox-LDL. The mRNA levels of VEGF and HIF-1*α* were detected by qPCR. Compared to the vehicle-treated group, intraperitoneal injection of A740003 significantly inhibited the mRNA levels of VEGF (38.07% ± 0.01256) (a) and HIF-1*α* (52.81% ± 0.01449) (b) in C57BL/6 mouse retina. Western blot showed that protein levels of VEGF (61.99% ± 0.01765) (c, d) and HIF-1*α* (59.28% ± 0.02902) (c, e) in C57BL/6 mouse retina were suppressed by A740003 significantly. Symbols: +: with; −: without. The results were mean ± SEM. Significance of difference (^∗^*p* < 0.05, ^∗∗^*p* < 0.01) was determined by using one-way ANOVA with Bonferroni correction (*n* = 4-6).

As previously described, C57BL/6 mice were subretinally injected with 1 *μ*l ox-LDL. A740003 (30 mg/kg body weight/day) was intraperitoneally injected daily from 3 days before ox-LDL injection. Two weeks later, qPCR and Western blot assay were performed to determine the mRNA and protein levels of VEGF and HIF-1*α*. As shown in Figure 5, mRNA levels of VEGF and HIF-1*α* were upregulated significantly in mice subretinally injected with ox-LDL (Figures 5(a) and 5(b)). Western blot results manifested that protein levels of HIF-1*α* and VEGF increased remarkably in mice subretinally injected with ox-LDL (Figures 5(c)–5(e)). It indicated that ox-LDL subretinal injection upregulated the angiogenic factors including VEGF and HIF-1*α* intensively, which would contribute to the retinal angiogenesis. In addition, Western blot results showed that protein levels of VEGF and HIF-1*α* decreased remarkably in mice treated with A740003 daily (Figures 5(c)–5(e)). Therefore, the results confirmed that the retinal angiogenic factors induced by ox-LDL were suppressed by A740003 treatment.

### 3.6. A740003 Inhibited Retinal Angiogenesis by Downregulating VEGF Expression In Vivo

**Figure 6:**
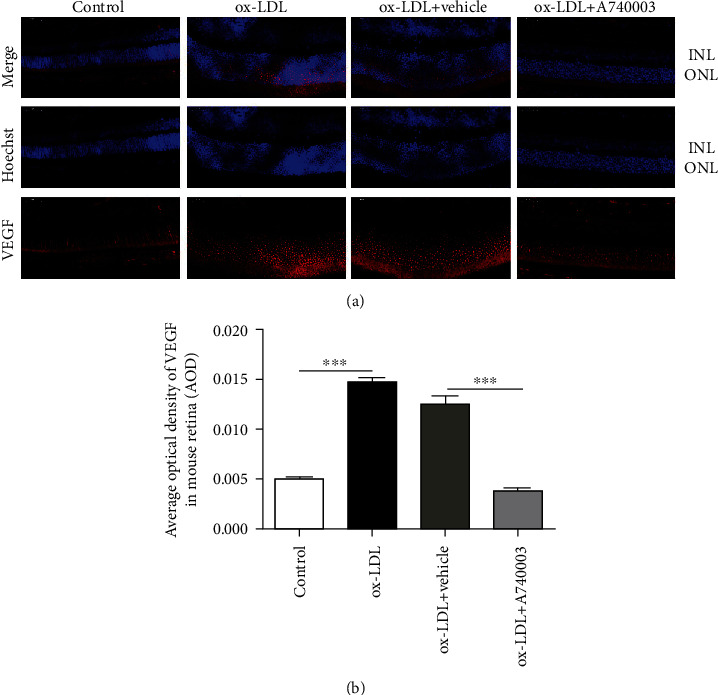
Protective effects of A740003 on the retinal angiogenesis of C57BL/6 mice induced by ox-LDL. The retinal angiogenesis in C57BL/6 mice was assessed by immunofluorescent staining of paraffin sections of whole eyes (a). Nuclei of cells in INL and ONL were stained by DAPI (blue; (a)) and VEGF was indicated by secondary antibody (red; (a)). The average optical density of VEGF (b) was summarized. Compared to the vehicle-treated group, A740003 decreased the VEGF expression significantly in the mouse retina induced by ox-LDL. Scale bar: 50 *μ*m. Abbreviations: INL: inner nuclear layer; ONL: outer nuclear layer. The results were mean ± SEM. Significance of difference (^∗^*p* < 0.05, ^∗∗^*p* < 0.01, and ^∗∗∗^*p* < 0.001) was determined by using one-way ANOVA with Bonferroni correction (*n* = 4-6).

**Figure 7:**
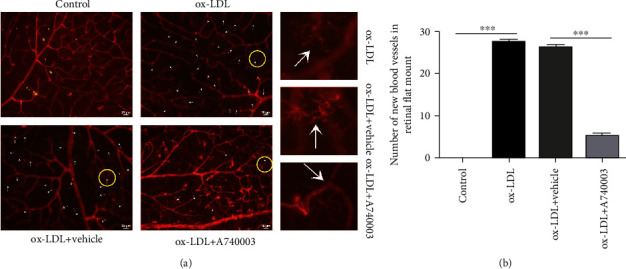
A740003 decreased the retinal new blood vessels of C57BL/6 mice induced by ox-LDL. The retinal angiogenesis in C57BL/6 mice was assessed by retinal whole flat mount, and vessels were stained by isolectin-B4 (red; (a)). The numbers of new blood vessels (arrows) in retinas were summarized (b). Compared to the vehicle-treated group, A740003 reduced the new blood vessels in retinas significantly. The results were mean ± SEM. Significance of difference (^∗∗∗^*p* < 0.001) was determined by using one-way ANOVA with Bonferroni correction (*n* = 4-6).

C57BL/6 mice were subretinally injected with ox-LDL and treated with A740003 daily from 3 days before ox-LDL injection. Two weeks later, immunofluorescent assay and retinal whole flat mount were processed to investigate the retinal angiogenesis in C57BL/6 mice. The results of immunofluorescent assay showed that ox-LDL increased the VEGF level robustly in retinas after two weeks of ox-LDL subretinal injection (Figures 6(a) and 6(b)). However, A740003 decreased the VEGF expression induced by ox-LDL significantly in mice compared to the vehicle-treated group (Figures 6(a) and 6(b)). The spontaneous retinal new vessels feature protrusions and outgrowths of capillary buds and sprouts [30]. Isolectin-B4 was used for staining retinal vessels. The retinal whole flat mount showed that ox-LDL successfully induced the retinal angiogenesis in mice (Figures 7(a) and 7(b)). However, A740003 decreased the number of new blood vessels in retinas significantly compared to the vehicle-treated group (Figures 7(a) and 7(b)). The results indicated that A740003 suppressed the retinal angiogenesis induced by ox-LDL successfully.

### 3.7. A740003 Prevented the Retinal Function Impairment Induced by ox-LDL In Vivo

**Figure 8:**
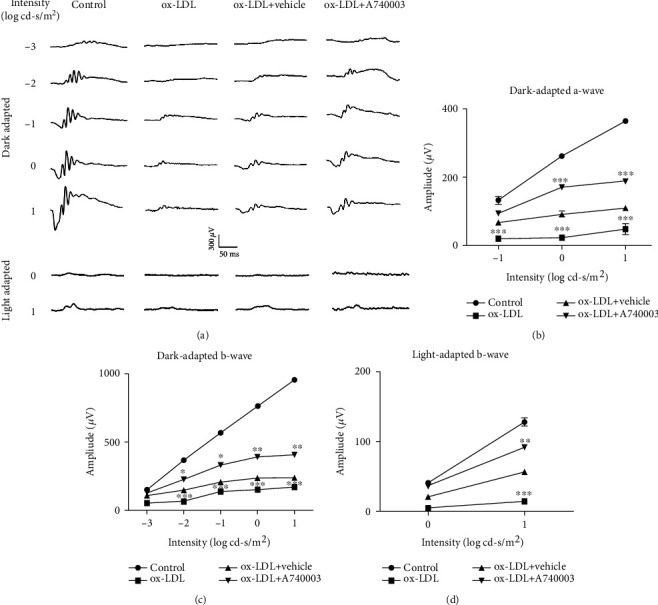
Retinal functions of C57BL/6 mice were assessed by dark- and light-adapted ERG. A740003 prevented the malfunction in C57BL/6 mouse retinas induced by ox-LDL. ERG was performed at 14 days after subretinal injection of ox-LDL. Representative ERG responses in the control, the ox-LDL, the ox-LDL plus vehicle-treated, and the ox-LDL plus A740003-treated groups (a) were shown. The ERG amplitudes vs. flash intensity profiles for the dark-adapted a-wave (b), dark-adapted b-wave (c), and the light-adapted b-wave (d) were summarized. Intraperitoneal injection of A740003 significantly preserved the ERG amplitudes compared to the vehicle-treated group. The results were mean ± SEM. Significance of difference (^∗^*p* < 0.05, ^∗∗^*p* < 0.01, and ^∗∗∗^*p* < 0.001) was determined by using two-way ANOVA with Bonferroni correction (*n* = 6-8).

C57BL/6 mice were pretreated with A740003 daily from 3 days before ox-LDL injection. Two weeks after subretinal injection of ox-LDL, ERG was performed to evaluate the retinal function of mice injected with ox-LDL or PBS. Dark- and light-adapted ERGs showed that the amplitudes of a-wave and b-wave, representing the functions of the photoreceptors and bipolar cells and Müller glial cells, respectively, decreased significantly in mice subretinally injected with ox-LDL (Figure 8(a)). However, the amplitudes of dark-adapted a-wave and b-wave in A740003 treated mouse retinas were higher than those in vehicle-treated mice (Figures 8(b) and 8(c)). The amplitude of light-adapted b-wave was also preserved by A740003 treatment (Figure 8(d)). It indicated that A740003 treatment could alleviate retinal function impairment induced by ox-LDL in mice.

## 4. Discussion

Accumulating evidence indicates that chronic inflammation in response to ox-LDL is implicated in the pathogenesis of AMD [8, 10, 25, 31]. In this study, we investigated the effects of ox-LDL on proinflammatory responses and proangiogenesis in human ARPE-19 cells and in a novel mouse model presenting inflammatory responses and neovascularization. We found that A740003, a P2X7 receptor antagonist, significantly inhibited ox-LDL-induced retinal inflammatory responses, neovascularization, and ROS generation and alleviated retinal function impairment in ox-LDL-treated cells and injected mice.

The results showed that ox-LDL induced proinflammatory responses and proangiogenesis. Besides, we further investigated the diverse effects of ox-LDL in ARPE-19 cells. Interestingly, we found that 24-hour incubation with ox-LDL could promote the inflammatory cytokines significantly in ARPE-19 cells, while the expression of HIF-1*α* and VEGF showed no significance. However, 48-hour incubation with ox-LDL could promote the expression of HIF-1*α* and VEGF in ARPE-19 cells significantly. It suggested that proangiogenesis occurred after the inflammatory responses, which mimicked the development of AMD where inflammation usually precedes neovascularization. In addition, ox-LDL also induced the production of ROS, which is involved in the process of AMD.

To confirm the role of ox-LDL in the retina, we established a mouse model by subretinal injection of ox-LDL. Similar to those findings observed *in vitro*, we found that IL-1*β*, HIF-1*α*, and VEGF levels were significantly increased in the mouse retinas 14 days after subretinal injection. Furthermore, retinal function was compromised and neovascularization was evident in ox-LDL-injected eyes. Since ox-LDL is a trigger for initiating early events in AMD and since the major pathologic processes including inflammatory responses and neovascularization were seen in the novel model, we suggested it might be an appropriate one for studying AMD.

To test whether P2X7R is involved in the pathogenesis of AMD and to test whether blocking P2X7R would prevent the proinflammatory responses and proangiogenesis induced by ox-LDL, A740003, a P2X7R antagonist, was applied to block P2X7R in human RPE cells and mouse model. The results demonstrated that A740003 decreased the secretion of inflammatory cytokines including IL-1*β* and IL-18 obviously both *in vitro* and *in vivo*. Moreover, the protein levels of VEGF and HIF-1*α* in A740003-treated group downregulated evidently compared with those in the vehicle-treated group both in cells and mouse retinas. Retinal immunofluorescence and retinal flat mount showed that A740003 alleviated the retinal neovascularization in ox-LDL-injected mice. It indicated that blocking P2X7R prevented the inflammatory response and angiogenesis induced by ox-LDL effectively.

P2X7R is an ATP ligand-gated nonselective cation channel that is a member of the purine receptor family. ATP could be synthesized and released by RPE cells in the mouse retina and ARPE-19 cells and activate P2X7R [32, 33]. We detected that ox-LDL promoted the ATP release, which in turn activated P2X7R (Figure S2). P2X7R activation leads to calcium ion influx and NLRP3 inflammasome activation [34]. P2X7R agonist induced the upregulation of proinflammatory mediators (IL-1*β*, etc.), P2X7R, and VEGF-A and enhanced the production of ROS [17]. The hypoxic NLRP3 and VEGF gene expression and the secretion of VEGF are in part mediated by purinergic receptor signaling [35]. NF-*κ*B and P2X7R are critical signaling intermediates in Alu RNA-induced inflammasome priming and RPE degeneration. P2X7R inhibitor protected against Alu RNA-induced RPE degeneration [36]. We detected the P2X7R expression in ARPE-19 cells and mouse retinas and discovered the relationship between P2X7R and NLRP3 inflammasome activation. Several concentrations of A740003 were screened for treatment in ARPE-19 cells (Figure S1). We found that 1 *μ*M A740003 reduced the expression of NLRP3, Caspase-1, and P2X7R significantly in ARPE-19 cells exposed to ox-LDL for 24 hours. However, 200 *μ*M A740003 was required to inhibit the expression of HIF-1*α* and VEGF increased with ox-LDL incubation for 48 hours. It suggested that it would be more and more difficult to reverse the harmful effects induced by ox-LDL with development. The underlying mechanisms of this phenomenon need further investigation. We also found that A740003 pretreatment reduced the upregulated protein level of P2X7R induced by ox-LDL. ox-LDL exposure increased P2X7R expression significantly in ARPE-19 cells. However, the protein level of P2X7R decreased remarkably in A740003-pretreated cells. Similar regulatory effects of P2X7R antagonist on P2X7R expression were ever reported [24].

The results showed that blocking P2X7R could decrease the protein levels of NLRP3, Capase-1, IL-1*β*, and P2X7R significantly both in ARPE-19 cells and mice. It revealed that A740003 suppressed the activation of NLRP3 inflammasome and alleviated the inflammation induced by ox-LDL. Moreover, we found that blocking P2X7R inhibited the phosphorylation of IKB*α* induced by ox-LDL significantly both in cells and mouse retinas. It indicated that A740003 could inhibit the activation of the NF-*κ*B pathway effectively.

We verified that a single subretinal injection of ox-LDL induced the activation of the NLRP3 inflammasome and NF-*κ*B signaling pathway in mice. Once NLRP3 inflammasome was activated, it would recruit ASC and mediate the autoactivation of pro-Caspase-1. The assembled NLRP3 inflammasome then turned itself into a cytokine processing platform by cleaving pro-IL-1*β*/pro-IL-18 into mature peptides and releasing them into extracellular space for downstream inflammatory effects [12]. In brief, ox-LDL produced a large amount of ROS and thus activated NLRP3 inflammasome and then promoted the secretion of inflammatory cytokines including IL-1*β* and IL-18. IL-1*β* and IL-18 are major inflammatory factors found in drusen of AMD patients [37].

ROS is an important trigger for cell pyroptosis. ROS could activate NLRP3 inflammasome and induce inflammation [12]. However, A740003 treatment inhibited the ROS production induced by ox-LDL significantly. It manifested that blocking P2X7R reduced the ROS overproduction. The underlying mechanisms of decrease in ROS production remain unknown. Moreover, ROS generation induced by ox-LDL can also lead to the activation of the NF-*κ*B signaling pathway. NF-*κ*B is a nuclear transcription factor that regulates the expression of a large number of genes that are critical for the regulation of apoptosis and inflammation. The activation of the NF-*κ*B signaling pathway could promote the transcription of NLRP3. The various stimuli that activate NF-*κ*B would cause phosphorylation of I*κ*B, which is followed by its ubiquitination and subsequent degradation [12].

The results indicated that ox-LDL induced retinal inflammation and neovascularization through activation of the NF-*κ*B pathway and NLRP3 inflammasome, induction of ROS generation, and upregulating the expression of HIF-1*α* and VEGF.

Although the study reveals strong evidence for the pathogenic roles of ox-LDL and therapeutic effects of A740003 in AMD, there are still some limitations that must be addressed. First, the direct interaction of P2X7R and NLRP3 needs further investigation, which may provide more details about the relationship between the activation of P2X7R and NLRP3 inflammasome. Moreover, further researches and trials need to be performed to testify the effectiveness and safety of P2X7R antagonist. There is still a long way to go before applying the P2X7R antagonist to AMD in clinic.

In conclusion, we demonstrated that A740003 significantly reduced inflammatory responses and angiogenic factors in ARPE-19 cells induced by ox-LDL. In addition, the intraperitoneal injection of A740003 prevented retinal inflammation and neovascularization and preserved retinal function in C57BL/6 mice subretinally injected with ox-LDL. The P2X7R antagonist could reduce retinal inflammation and neovascularization and protect retinal function by regulation of the NLRP3 inflammasome and NF-*κ*B pathway and suppression of ROS generation, as well as inhibition of HIF-1*α* and VEGF [38]. The results provide a new clue for therapeutic strategies to treat retinal inflammation and neovascularization.

## Figures and Tables

**Table 1:** Sequence of primers for human.

Gene	Forward primer	Reverse primer
NLRP3	GATCGTGAGAAAACCCTCCA	GGTCCTATGTGCTCGTCAAA
P2RX7	AGGAAGAAGTGCGAGTCCATTGTG	CTGAACAGCTCTGAGGTGGTGATG
VEGFA	CAGATTATGCGGATCAAACCT	ACGTTCGTTTAACTCAAGCT
HIF1A	GAACGTCGAAAAGAAAAGTCTCG	CCTTATCAAGATGCGAACTCACA
ACTB	GCCAACCGCGAGAAGATGACC	CTCCTTAATGTCACGCACGATTTC

**Table 2:** Sequence of primers for mouse.

Gene	Forward primer	Reverse primer
Nlrp3	CTCTGTTCACTGGCTGCGGATG	TAGGACCTTCACGTCTCGGTTCAG
P2rx7	GCATAGCAGAGGTGACGGAGAATG	AGTAGGACACCAGGCAGAGACTTC
Vegfa	CGAAGCTACTGCCGTCCGATTG	CCGCTCTGAACAAGGCTCACAG
Hif1a	ACCTTCATCGGAAACTCCAAAG	CTGTTAGGCTGGGAAAAGTTAGG
Casp1	CGTGGAGAGAAACAAGGAGTG	AATGAAAAGTGAGCCCCTGAC
Actb	TCACTATTGGCAACGAGCGGTTC	CTCCTGCTTGCTGATCCACATCTG

## Data Availability

The data used to support the findings of this study are included within the article.

## Supplementary Materials

Supplementary Materials Figure S1: the dose screening of A740003 treatment in ARPE-19 cells. Figure S2: ox-LDL exposure increased the ATP content in ARPE-19 cells.
