# Supplementary material for: P2X7 Receptor Antagonist Attenuates Retinal Inflammation and Neovascularization Induced by Oxidized Low-Density Lipoprotein
Source: Oxid Med Cell Longev. 2021 Aug 19;2021:5520644. doi: 10.1155/2021/5520644 (PMC8397555; doi:10.1155/2021/5520644)
Supplement: Supplementary Materials — Figure S1: the dose screening of A740003 treatment in ARPE-19 cells. Figure S2: ox-LDL exposure increased the ATP content in ARPE-19 cells. [file 5520644.f1.docx]

Supplementary Materials：


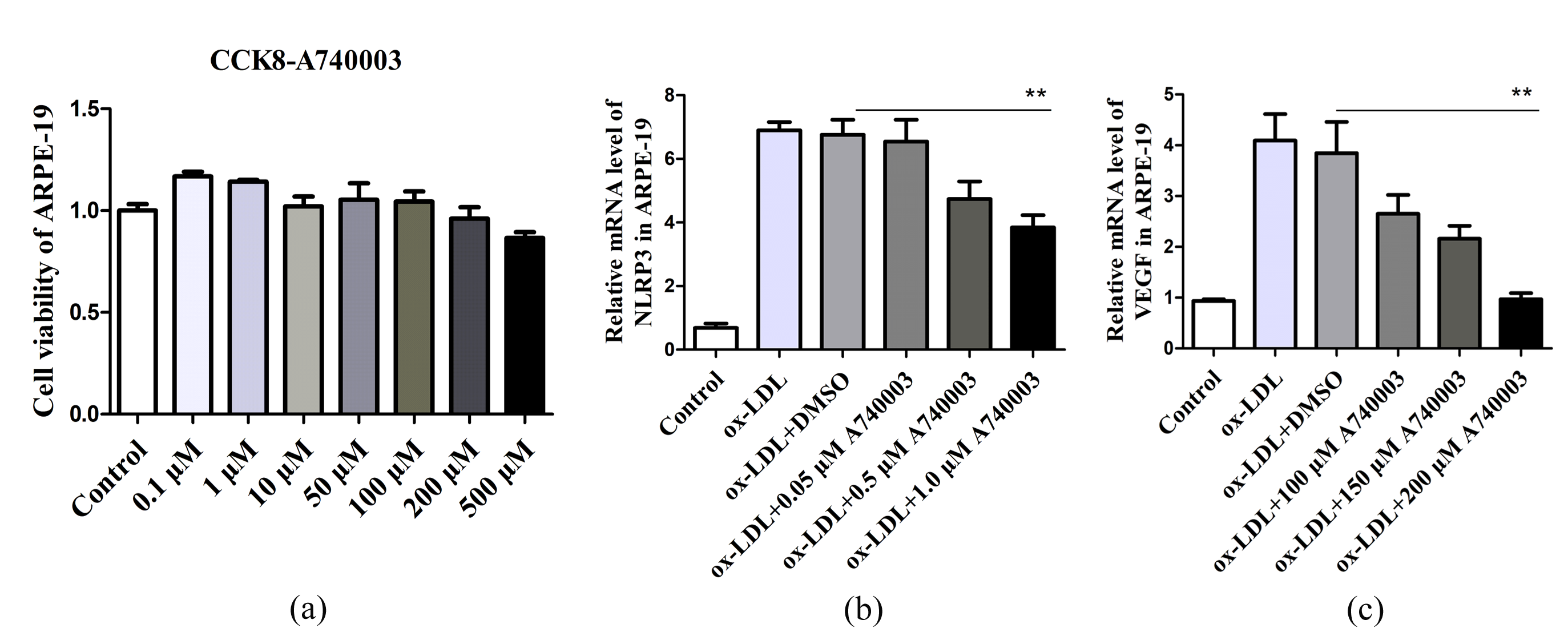


FIGURE S1: The dose screening of A740003 treatment in ARPE-19 cells. 0.1 μM to 500 μM A740003 were screened for A740003 cytotoxicity by using CCK8 assay (a). The optimal concentration of A740003 for anti-inflammation effects was evaluated by NLRP3 expression (b). The optimal concentration of A740003 for anti-angiogenesis effects was determined by VEGF expression (c). The results were mean ± SEM. Significance of difference (***p*<0.01) was determined by using one-way ANOVA with Bonferroni correction.


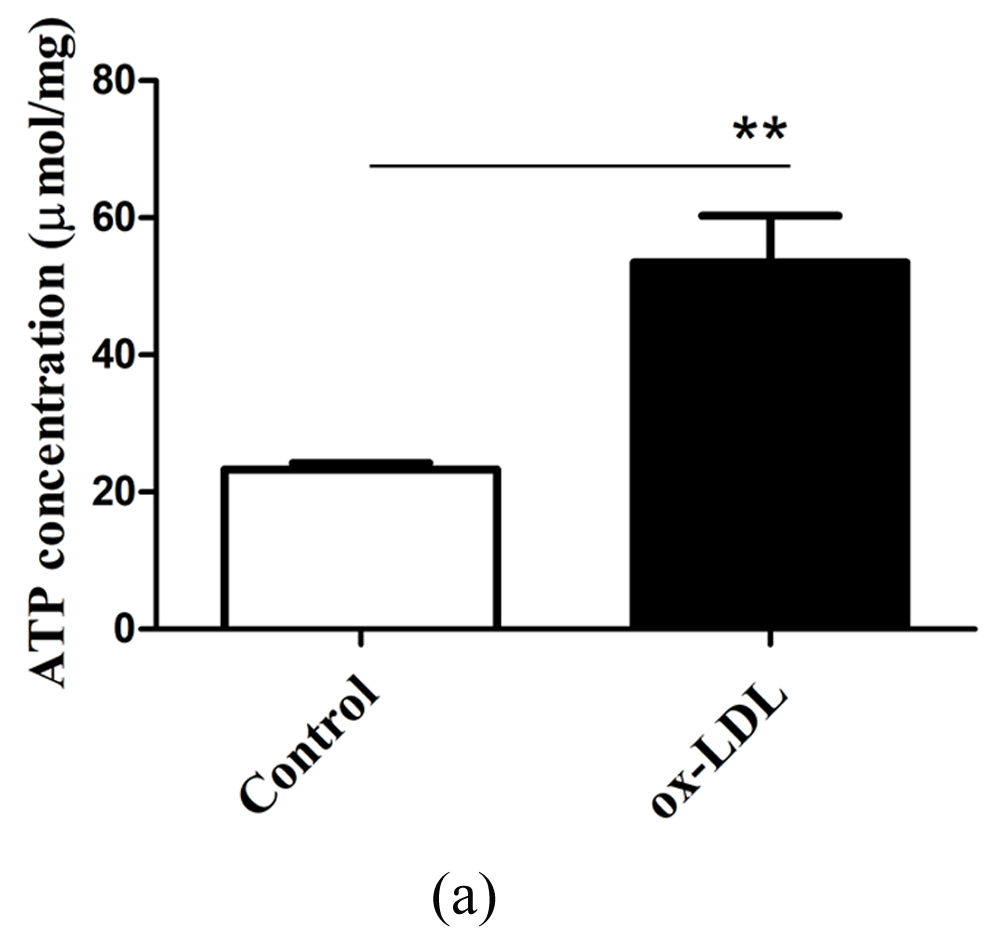


FIGURE S2: Ox-LDL exposure increased the ATP content in ARPE-19 cells. The fluorescence indicating ATP level increased robustly in ARPE-19 cells incubated with ox-LDL (a). The results were mean ± SEM. Significance of difference (**p<0.01) was determined by using Student’s t-test.
